# Quantifying and mathematical modelling of the influence of soluble adenylate cyclase on cell cycle in human endothelial cells with Bayesian inference

**DOI:** 10.1111/jcmm.17611

**Published:** 2022-11-13

**Authors:** Warunya Woranush, Mats Leif Moskopp, Thomas Noll, Peter Dieterich

**Affiliations:** ^1^ Institut für Physiologie, Medizinische Fakultät Carl Gustav Carus, Technische Universität Dresden Dresden Germany; ^2^ Vivantes Klinikum im Friedrichshain, Charité Academic Teaching Hospital, Klinik für Neurochirurgie Berlin Germany

**Keywords:** ADCY10, Bayesian inference, cell cycle, cell proliferation, mathematical modelling

## Abstract

Adenosine‐3′, 5′‐cyclic monophosphate (cAMP) produced by adenylate cyclases (ADCYs) is an established key regulator of cell homoeostasis. However, its role in cell cycle control is still controversially discussed. This study focussed on the impact of soluble HCO_3_^−^ ‐activated ADCY10 on cell cycle progression. Effects are quantified with Bayesian inference integrating a mathematical model and experimental data. The activity of ADCY10 in human umbilical vein endothelial cells (HUVECs) was either pharmacologically inhibited by KH7 or endogenously activated by HCO_3_^−^. Cell numbers of individual cell cycle phases were assessed over time using flow cytometry. Based on these numbers, cell cycle dynamics were analysed using a mathematical model. This allowed precise quantification of cell cycle dynamics with model parameters that describe the durations of individual cell cycle phases. Endogenous inactivation of ADCY10 resulted in prolongation of mean cell cycle times (38.7 ± 8.3 h at 0 mM HCO_3_^−^ vs 30.3 ± 2.7 h at 24 mM HCO_3_^−^), while pharmacological inhibition resulted in functional arrest of cell cycle by increasing mean cell cycle time after G_0_/G_1_ synchronization to 221.0 ± 96.3 h. All cell cycle phases progressed slower due to ADCY10 inactivation. In particular, the G_1_‐S transition was quantitatively the most influenced by ADCY10. In conclusion, the data of the present study show that ADCY10 is a key regulator in cell cycle progression linked specifically to the G_1_‐S transition.

## 1 INTRODUCTION

Endothelial cells play a key role in angiogenesis. Physiological angiogenesis is a driving force of embryogenesis or cardiovascular regeneration.^1^, ^2^ By contrast, under pathophysiological conditions, it causes neovascularization during tumour formation and metabolic disease such as diabetes mellitus.^3^, ^4^ However, angiogenesis is known to be driven by the metabolic state of the microenvironment of endothelial cells. To preserve a pH‐stable microenvironment, bicarbonate (HCO_3_^−^) acts as a ubiquitous endogenous buffer. Physiological bicarbonate concentrations in mammalian blood range between 22 and 26 mM (corresponding to 5% [v/v] CO_2_ typically used in cell culture incubators), while peak values under metabolic load in the venous blood of the human heart can reach up to 40 mM.^5^

Adenylate cyclases (ADCYs) catalyse the substrate ATP to cAMP.^6^, ^7^ ADCY10 is the only soluble isoform of the otherwise transmembrane adenylate cyclases (ADCY1‐9). While ADCY1‐9 are activated by external stimuli via G protein‐coupled receptors (GPCRs), the activity of ADCY10 depends on the intracellular bicarbonate (HCO_3_^−^) concentration.^8^, ^9^ Therefore, ADCY10 acts as an endogenous metabolic sensor that produces the second messenger cAMP.^10^, ^11^

cAMP plays a central role in multiple intracellular pathways such as the regulation of cell homoeostasis, metabolism, and cell growth.^12^, ^13^, ^14^, ^15^, ^16^, ^17^, ^18^ Although multiple studies focussed on the role of cell type‐specific effects of cAMP on cell cycle,^19^, ^20^, ^21^, ^22^, ^23^, ^24^, ^25^, ^26^, ^27^ most of these studies established a detailed understanding of the influence of the transmembrane isoforms ADCY1‐9. However, ADCY10 was found to localize in different intracellular compartments such as the nucleus.^28^, ^29^ Further, ADCY10 is shown to be colocalized with mitotic spindles during mitosis suggesting a distinct role in cell cycle regulation.^29^ Nevertheless, its quantitative influence on the different stages of cell cycle progression has not been studied in detail.^30^ Here, we used human umbilical vein endothelial cells (HUVECs) as a model to study the influence of ADCY10 on cell cycle.^31^, ^32^, ^33^

Cell cycle phases were experimentally assessed using flow cytometry. To quantify and interpret experimental data, we suggest a mathematical model that describes temporal cell cycle progression based on a set of coupled differential equations allowing to quantify the transition rates between the individual cell cycle phases by four parameters. Moreover, we simulate in silico data for experimental planning to estimate the necessary amount of data for analysis, resulting in efficiently timed workload for the experimenter and cost‐effective usage of laboratory materials. Thereby, Bayesian inference allows a logically consistent and reliable estimation of parameters and their uncertainties.

ADCY10 is one among few known enzymes, which is directly activated by bicarbonate. In principle, ADCY10 has three known activators, which are ATP, Ca^2+^ and HCO_3_^−^. Obviously, Ca^2+^ and ATP concentrations are not feasible for cell cycle proliferation studies (e.g. elevated intracellular Ca^2+^ concentrations would lead to apoptosis). To particularize the role of ADCY10, we used different bicarbonate concentrations as well as the specific inhibitor KH7.

It is the central aim of this study to identify how ADCY10 influences the timing of the individual phases of the cell cycle in HUVECs. A mathematical model‐based technique is applied that allows for experimental planning and in‐depth analysis of flow cytometry‐based time course studies of in vitro data. The main biological result emerging from this analysis is that bicarbonate‐dependent ADCY10 activation in human endothelial cells affects all cell cycle phases and controls the duration of G_1_ phase in particular.

## 2 MATERIALS AND METHODS

### 2.1 Biological experiments

**FIGURE 1:**
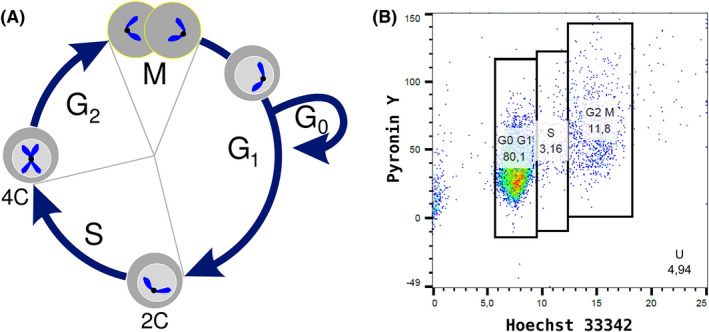
(A) Cell cycle stages of a somatic cell. Cell cycle stages consist of two major phases: Interphase (G_1_, S, G_2_) and mitotic phase (M). During gap/growth phase 1 (G_1_) cells prepare for DNA synthesis in S phase, while cells in gap/growth phase 2 (G_2_) prepare for cell division during mitotic phase (M). Additionally, cells can stay quiescent in G_0_ and later reenter the cell cycle. DNA content is described as follows: 2C: single diploid chromosome set, 4C: double diploid chromosome set. (B) Flow cytometry enables the differentiation between cell cycle phases based on DNA content in G_0_/G_1_, S, and G_2_/M. Numbers represent percentages of cells in each phase. The graph demonstrates that in general around 80% of the cells are in G_0_/G_1_ after cell synchronization due to starvation up to 12 h after cell desynchronization. The variable U records the percentage of unclassified particles and cell debris. Hoechst 33342 and Pyronin are a DNA and RNA staining, respectively.

Figure 1 illustrates the experimental assessment of cell cycle phases.

#### 2.1.1 Isolation and culture

HUVECs were isolated from fresh umbilical cords (Ethics approval, cf. Appendix S1: Supplement S1) according to Jaffe et al. (1973) with some modifications.^34^ Consecutively, cells were seeded into T25 flasks and allowed to attach overnight. Cells were washed with phosphate‐buffered saline (PBS) and allowed to grow in M199 media (Sigma, Missouri, USA) supplemented with 2% foetal calf serum (FCS), supplement pack endothelial cell growth medium (PromoCell, Heidelberg, Germany), 1 mM sodium pyruvate (Sigma, Missouri, USA), 2 mM L‐glutamine (Sigma, Missouri, USA), and 25 mM HEPES (Sigma, Missouri, USA) at 37 °C in 5% (v/v) CO_2_ until reaching confluency. Afterwards, primary cells were trypsinized and used for cell proliferation experiments.

#### 2.1.2 Cell proliferation assay

HUVECs were seeded on 3.5 cm^2^ culture dish (Corning™ Falcon™, New York, USA) with a density of 50,000 cells/dish in M199 medium supplemented with 2% FCS for 24 hours (h). Afterwards, cells were washed with 1X Hanks' balanced salt solution (HBSS) to remove residue of FCS followed by adding M199 medium supplemented with 0.2% FCS and incubated for 18 h. This adapted method^35^ was used to induce cell synchronization in G_0_/G_1_ phases. After 18 h of cell synchronization, two sets of experiments were conducted. In set A, HUVECs were exposed to 0, 24, or 40 mM HCO_3_^−^, corresponding to 0, 5, or 8% (v/v) CO_2_, in M199 media supplemented with 10% FCS containing 50 mM HEPES (Sigma, Missouri, USA), at pH 7.4. In set B, HUVECs were treated with 10 μM ADCY10 inhibitor KH7 (CAYMAN chemical, Michigan, USA) dissolved in dimethyl sulfoxide (DMSO) as vehicle according to Bitterman et al.^9^ whereas the control group obtained DMSO only. Both groups were stored in an incubator at 24 mM HCO_3_^−^ in M199 medium supplemented with 10% FCS containing 25 mM HEPES at pH 7.4. In both sets of experiments, HUVECs were allowed to proliferate for up to 72 h. During set A experiments, cells were trypsinized and collected every 12 h (12, 24, 36, 48, 60, and 72 h). Consecutively, cells were fixed and proceeded to flow cytometry to quantify cell number and cell cycle stages. Experiments involving KH7 were performed in an identical procedure as for set A, except that cells were collected at 12, 24, 48, and 72 h.

#### 2.1.3 Flow cytometry

Cell number and cell cycle stages were assessed as described by Kim and Sederstrom (2015).^36^ Briefly, HUVECs were trypsinized and then washed with 1X PBS. Subsequently, HUVECs were fixed with 70% ethanol overnight. The cell pellets were then stained with freshly prepared 2 μg/mL Hoechst 33342 (Sigma, Missouri, USA) and 4 μg/mL pyronin Y (Sigma, Missouri, USA) staining solution. Consecutively, the resuspended stained cells were pipetted into a flat bottom 96‐well plate (Greiner Bio‐One GmbH, Frickenhausen, Germany) and analysed by a flow cytometer MACSQuant Analyzer 10 (Miltenyi Biotec GmbH, Bergisch Gladbach, Germany). Data obtained from flow cytometry were processed and analysed using FlowJo^™^ Software (FlowJo LLC, Ashland, Oregon, USA). The data are available via the zenodo archive.^37^

### 2.2 A mathematical model of the cell cycle

**FIGURE 2:**
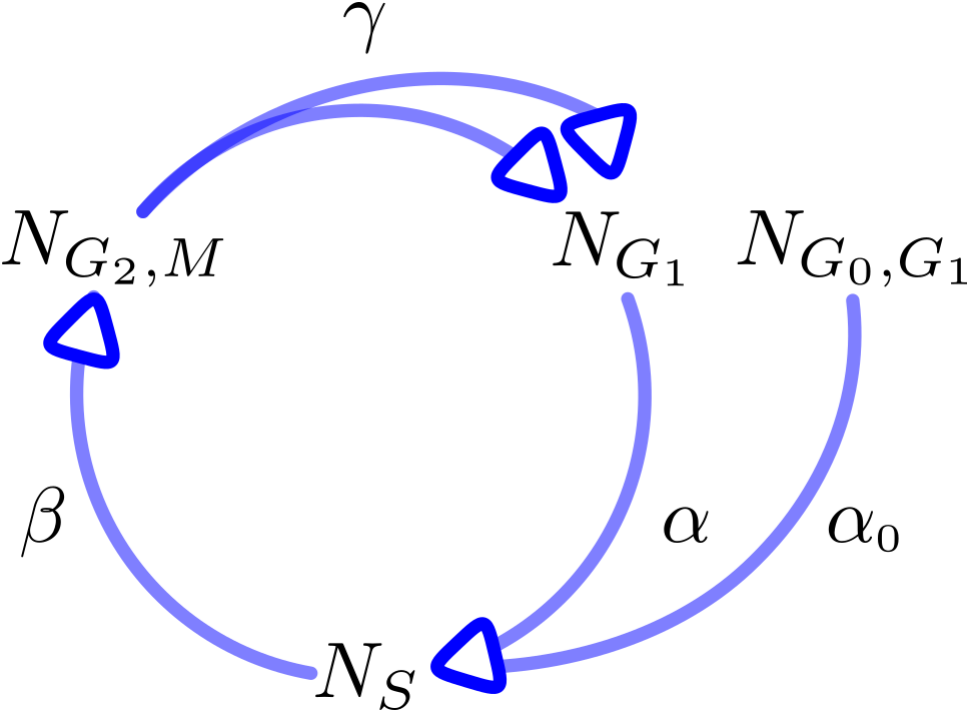
Schematic flow chart of the underlying mathematical model. The model describes the cell cycle progression after serum starvation and cell cycle arrest in G_0_ as a function of time. Cells are in one of the following states G_0_/G_1_, G_1_, S and G_2_/M. The amount of cells in one of the states at any given time point t is labeled N_G0,G1_(t), N_G1_(t), N_S_(t) and N_G2,M_(t) respectively. Since resident times of cells in G_1_ depend on whether they enter from M or G_0_ phase, these entities are summarized as either N_G1_ or N_G0,G1_.^37^ The arrows indicate transitions between these states. Transition rates are described by the variables α, α_0_, β and γ.

A mathematical model represents a formal description of main components of the observed process, while the model parameters allow a quantitative description. Figure 2 illustrates the mathematical model, which was used in this work as a flow chart. The variables N_G0,G1_(t), N_G1_(t), N_S_(t) and N_G2,M_(t) describe the absolute number of cells in the individual phases at time point t. Furthermore, the variable N(t) describes the total cell number. Additionally, the variables n_G0,G1_(t), n_G1_(t), n_S_(t) and n_G2,M_(t) describe the percentage of cells in a certain state. We assume that cells can enter and leave all cell cycle phases except for G_0_, which does not allow for cell entry, since we assume that the number of cells entering G_0_ in nutrition‐rich media after a period of extended starvation is neglectable. Transition from one phase to another is assumed as proportional to the number of cells in the preceding phase. The model parameters α, α_0_, β and γ represent the transition rates between cell cycle phases. Transition rates are assumed to be constant throughout the observation period. While, for example, α describes the probability rate for cells to transit from G_1_ to S phase, the reciprocal 1/α describes the mean residence time for cells to stay in G_1_ phase.^38^

Formally, the mathematical model is given by a set of coupled, first‐order linear ordinary differential equations (ODE):

(Eq 1) $$\frac{d}{\mathrm{dt}}N_{G0,G1}\left(t\right)=-\alpha_{0}\times N_{G0,G1}\left(t\right)$$

(Eq 2) $$\frac{d}{\mathrm{dt}}N_{G1}\left(t\right)=2\times \gamma \times N_{G2,M}\left(t\right)-\alpha \times N_{G1}\left(t\right)$$

(Eq 3) $$\frac{d}{\mathrm{dt}}N_{S}\left(t\right)=\alpha \times N_{G1}\left(t\right)+\alpha_{0}\times N_{G0,G1}\left(t\right)-\beta \times N_{S}\left(t\right)$$

(Eq 4) $$\frac{d}{\mathrm{dt}}N_{G2,M}\left(t\right)=\beta \times N_{S}\left(t\right)-\gamma \times N_{G2,M}\left(t\right)$$

(Eq 5) $$\frac{d}{\mathrm{dt}}N\left(t\right)=\gamma \times N_{G2,M}\left(t\right)$$

Equation 5 represents the temporal development of the total cell number N(t) as the sum of Equation (Eq 1), (Eq 2), (Eq 3), (Eq 4). Due to cell proliferation caused by parameter γ, this system does not reach a steady state. However, the percentage of cells in a certain state, for example, n_G0,G1_(t) = N_G0,G1_(t)/N(t) converges towards a steady state for long times (cf. Appendix S1: Supplement S2).

The mathematical model described above is deterministic for a given set of initial conditions at time t_0_ including N(t_0_), N_G0,G1_(t_0_), N_G1_(t_0_), N_S_(t_0_) and N_G2,M_(t_0_) and transition rates, consisting of α, α_0_, β and γ. To facilitate calculations, n(t_0_) is set to 100% and, respectively, converted in the data. Since flow cytometry measurements include counts of debris, aggregates or staining defects, the raw classified cell numbers of the individual phases of the cell cycle may not add up to 100% within one experimental measurement. Thus, an additional parameter U is used to record these unclassified counts, which are consecutively excluded from further analysis (cf. Appendix S1: Supplement S3). A parameter set containing all of these nine free model parameters is denoted θ throughout this work (Table 1).

**TABLE 1:** Free model parameters

Name	Unit	Explanation
Start conditions		
N_G1_(t_0_)		Initial cells in G_1_ phase
N_G0,G1_(t_0_)		Initial cells in G_0_/G_1_ phase
N_S_(t_0_)		Initial cells in S phase
N_G2,M_(t_0_)		Initial cells in G_2_/M phase
Model parameters		
α	(1/h)	Transition rate from G_1_ to S
α_0_	(1/h)	Transition rate from G_0_G_1_ to S
β	(1/h)	Transition rate from S to G_2_/M
γ	(1/h)	Transition rate from G_2_/M to G_1_
Unclassified count estimator		
U	(%)	Unclassified counts. Takes into account that experimental flow cytometry data does not always add up to 100%.

Our mathematical model is a continuous, deterministic description of the cell cycle based on ODEs. Alternatively, discrete stochastic models could be applied to simulate the individual state of each.^39^ This approach might facilitate model perception in an interdisciplinary collaboration, but it is computationally more elaborate. However, we show with simulations that both approaches agree under the assumption that cell cycles display exponential waiting time distributions (cf. Appendix S1: Supplement S4, Figure SF1). These distributions are characterized by the rate constants of the continuous model.

### 2.3 Bayesian inference in data analysis and numerical implementation

Bayesian analysis allows the estimation of model parameters given the amount and the quality of experimental data. In this context, Bayesian inference is a logically consistent approach that takes measurement uncertainties into account and thus allows a reliable estimation of the means and uncertainties of model parameters.

In general, the Bayesian theorem is used to calculate the conditional probability P(A|B) for event A given that event B occurred.

(Eq 6) $$P\left(A|B\right)=\frac{P\left(B׀A\right)\times P\left(A\right)}{P\left(B\right)}$$

Here, the probabilities P(A) and P(B) describe the unconditional probabilities for events A and B, while the term P(B|A) describes the conditional probability of event B given that event A is true.

In terms of data processing and mathematical modelling, the Bayesian theorem can be used to create a link between model parameters and experimental data.^40^, ^41^, ^42^, ^43^ The conditional probability P(θ|D) for a parameter set θ, representing the J free model parameters (θ_1_, θ_2_, …, θ_J_), and given the observed biological experimental data D can be expressed as:

(Eq 7) $$P\left(\theta |D\right)=\frac{P\left(D׀\theta \right)\times P\left(\theta \right)}{P\left(D\right)}$$

The term P(θ) is called *prior* in Bayesian terminology. It summarizes information about the parameter values based on literature data or prior knowledge but not based on any data of the current analysis.

The conditional probability P(D|θ) is called likelihood (LH) in Bayesian terminology. It creates a close link between the data and the mathematical model containing the parameters. The likelihood is a measure of how likely every experimental data point d_i_ of the experimental data D (with I data points d_i_, i = 1 … I) can be explained by a data point m_i_ of the mathematical model for a given parameter set θ. Under the assumption of normally distributed measurement errors σ_i_ and independently recorded data points, the likelihood can be written as a product of Gaussian likelihoods for all data points^40^, ^41^:

(Eq 8) $$\mathrm{LH}=P\left(D|\theta \right)=\prod_{i=1}^{I}\frac{1}{\sqrt{2\pi \sigma_{i}^{2}}}\times \exp\left(-\frac{1}{2}\frac{{\left(d_{i}-m_{i}\left(\theta \right)\right)}^{2}}{\sigma_{i}^{2}}\right)$$

The conditional probability P(θ|D) is called posterior in Bayesian terminology. It describes the probability density function of all parameters given the observed experimental data. The posterior is one main result of the parameter estimation. The estimated posterior probability density function marginalized for an individual parameter θ_k_ is obtained by integration of all other parameters θ_j_ with k ≠ j

(Eq 9) $$P\left(\theta_{k}|D\right)=\int \prod_{\begin{array}{c} j=1\\ j\neq k \end{array}}^{J}d\theta_{j}P\left(\theta_{1},\theta_{2},\ldots ,\theta_{J}|D\right)$$

and will typically be represented by its mean and standard deviation (Bayesian uncertainty, bu, cf. Appendix S1: Supplement S5, Table ST1). Finally, the term P(D) of Equation 7 is called Bayesian evidence, which formally normalizes the posterior.

Here, we used the Python interface pymultinest of the multinested extension of the nested sampling algorithm as a numerical implementation of Bayesian inference to obtain the marginalized posteriors of each parameter from Equation 9 (cf. Appendix S1: Supplement S6 and S7).^44^, ^45^, ^46^

### 2.4 Statistical test

Significance among groups was determined using one‐way analysis of variance anova followed by Student–Newman–Kuel test (GraphPad Inplot, ISI software or PRISM). Changes in parameters within the same group were assessed by multiple anova analysis or paired t‐Test. Probability values (*p*‐values) of less than 0.05 were considered significant (*p* < 0.05) (cf. Appendix S1: Supplement S8).

## 3 RESULTS

### 3.1 Cell cycle analysis based on raw flow cytometry data

**FIGURE 3:**
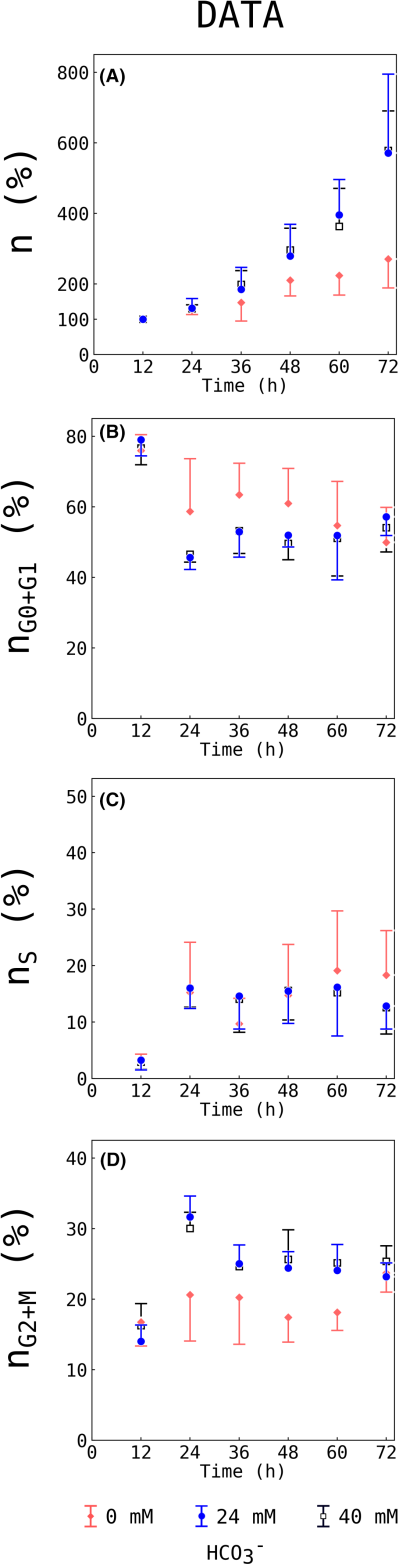
Effect of bicarbonate deprivation on HUVEC experimental data. Flow cytometry was used to measure the total number (A) and percentage of cells in the individual cell cycle phases (B‐D) as a function of time for bicarbonate concentrations of 0 (♦, red), 24 (●, blue) and 40 (□, black) mM. Experimental data is shown with mean ± SD. Based on DNA amount and flow cytometry cells in G_0_ and G_1_ as well as cells in G_2_ and M phase could not be differentiated and are therefore displayed as sum (n_G0+G1_, n_G2+M_). Experimental data was obtained from 5 independent experiments, with two replicates each.

Cells were experimentally either exposed to HCO_3_^−^ levels of 0 mM (deprivation), 24 mM (physiological) or 40 mM (supraphysiological) and subsequently analysed using flow cytometry. Figure 3 shows experimental data of cell proliferation and percentage in the different cell cycle phases as a function of time. 24 mM HCO_3_^−^ (control) showed increased cell proliferation compared to 0 mM (cf. Figure 3A). Experimental data showed no difference in cell cycle or total cell number between 24 mM and 40 mM at any given time point. Regarding cell cycle phases, all three experimental groups appear to reach equilibrium states with almost constant fractions of cells per phase. This is in accordance with cell cycle desynchronization. Cell fractions in G_0_/G_1_ dropped by around 35% between 12 and 24 h at 24 mM HCO_3_^−^ compared to 20% at 0 mM. As shown in Figure 3B, the fraction of cells in G_0_/G_1_ reaches a steady state faster at 24 mM HCO_3_^−^ compared to 0 mM HCO_3_^−^ suggesting a shorter residence period within this phase. However, a direct quantification of this observation is hardly possible from the raw flow cytometry data.

There was no difference in cell number in S phases at 24 h (cf. Figure 3C). Cell numbers increased in G_2_/M phases by 15% between 12 and 24 h at 24 mM HCO_3_^−^ compared to 5% at 0 mM HCO_3_^−^ (cf. Figure 3D).

In conclusion, the interpretation of the effect of HCO_3_^−^ on cell cycle dynamics is difficult to assess from raw flow cytometry data. In order to obtain deeper insights into the effects of ADCY10 on cell cycle dynamics, Bayesian inference was used to analyse biological data obtained by flow cytometry.

### 3.2 Quantifying information contained in flow cytometry data

Before applying model Equation (Eq 1), (Eq 2), (Eq 3), (Eq 4), (Eq 5) to experimental data, we determined with simulations the amount and quality of data to obtain a robust estimation of all nine model parameters. The following two research questions were addressed:

1. Which time intervals of flow cytometry time course studies are necessary to obtain precise parameter estimations for HUVECs?
2. Can correct parameter values be estimated if either the cell number N(t) or the cell cycle stages (n_G0,G1_(t), n_G1_(t), n_S_(t) and n_G2,M_(t)) were used separately for analysis?

Synthetic test data with known parameters (cf. Appendix S1: Supplement S9 and S10 and supplement Table ST3) mimicking HUVEC cell cycle progression were analysed. Test data were simulated with varying observation intervals dt = 1, 2, 4, 6, 12, 24, 36 h (cf. Appensix S1: Supplement S1), where smaller intervals generated more data. Analyses were performed for cell numbers only, cell cycle stages only and the combination of both for the individual data sets with varying dt, for example, varying the amount of available data.

**FIGURE 4:**
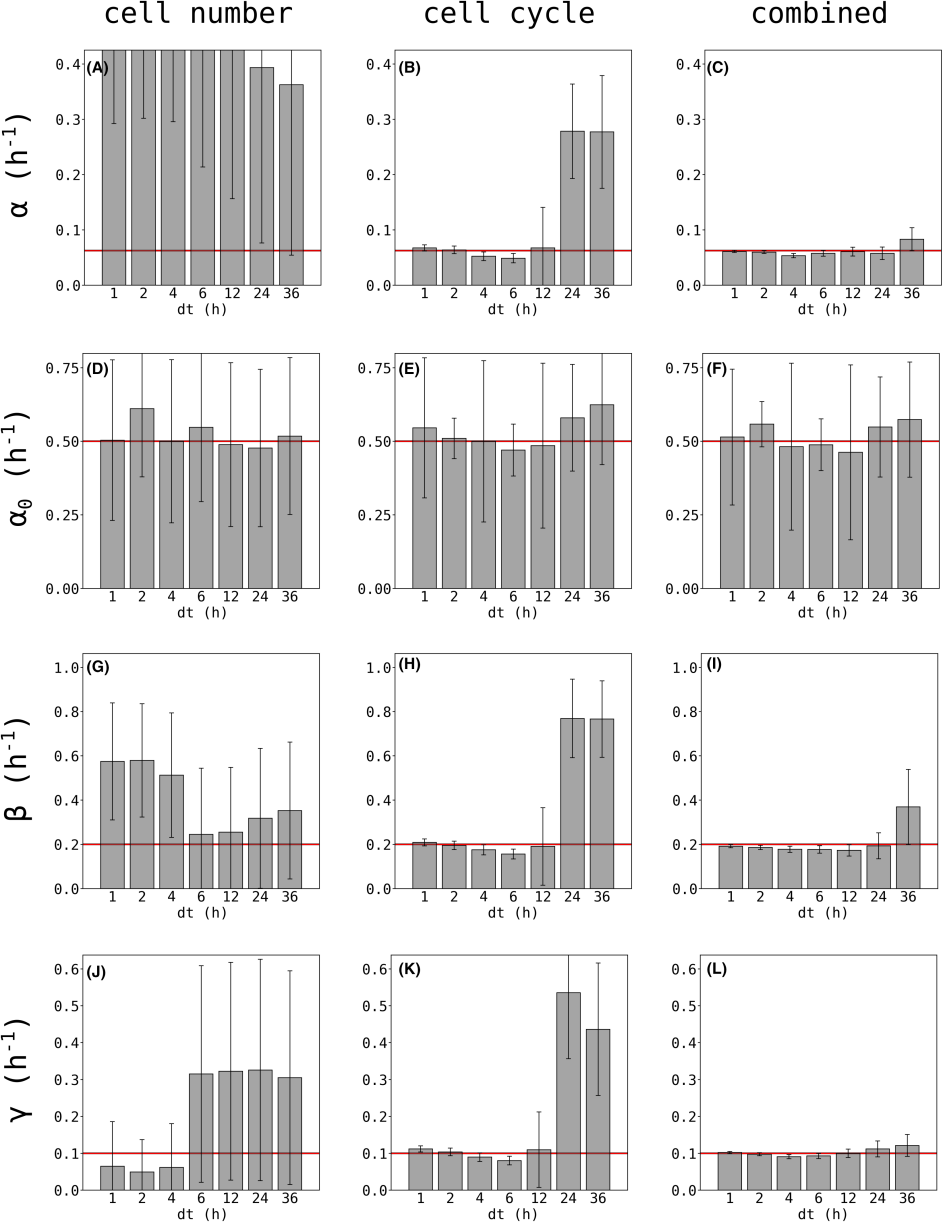
Parameter estimation of α, α_0_, β and γ for synthetic test data. Synthetic test data with a known set of parameters was generated according to Appendix S1: Supplement S4. Test data mimicked experimental observations of HUVECs. Analysis for different time intervals dt was performed based on cell numbers only (A, D, G, J), cell cycle stages only (B, E, H, K) or the combination of both of them (C, F, I, L). Bar graphs show estimated parameter values with mean and uncertainty. Red lines mark input values used for test data generation (Appendix S1: Supplementary S4, Table ST1). Highest overall precision was reached when cell number and cell cycle stages were analyzed together (right column).

Figure 4 shows the estimated mean values of parameters and their uncertainties. For the analysis of cell numbers alone (cf. Figure 4 left column), mean values of parameter showed large error bars and did not represent the synthetic input parameters well (see Appendix S1: Supplement S9, Table ST3 and red lines in Figure 4). The combined analysis of cell number and cell cycle phases increased the precision of parameter estimation (cf. Figure 4 right column). Generally, parameter estimation depended on sampling intervals dt, with smaller sampling intervals (corresponding to more data) resulting in smaller uncertainties. Based on these simulated data, experiments were performed with a sampling interval of 12‐h balancing workload and precision of parameter estimation.

As a side note, we want to point out the difference between cell cycle time and doubling time: While the ground truth for the mean cell cycle time (G_1_ + S + G_2_M) is 31 h, the Bayesian estimate for the test data set with observation interval dt = 12 h is 32.28 ± 2.63 h, which we considered adequate. In comparison with the cell cycle time, the doubling time is not a Bayesian estimate, but calculated according to equation SE7 (cf. Appendix S1: Supplement S11, equation SE7). The ground truth for the doubling time between 24 and 72 h is 27.04 h, while the calculation results in 28.20 h.

Thus, this pre‐analysis based on simulated test data showed the validity of model parameter estimation and additionally allowed an experimental design of the sampling interval of the performed experiment (cf. Appendix S1: Supplement S9, S10 and S12).

### 3.3 Effects of HCO_3_ ^−^ concentrations on HUVEC proliferation and cell cycle progression

Based on the biological experimental data, analyses were performed to investigate the influence of HCO_3_^−^ on HUVEC cell cycle progression.

#### 3.3.1 Model fits capture the dynamics of experimentally recorded cell cycle transition and identify extreme points of cell cycle dynamics

**FIGURE 5:**
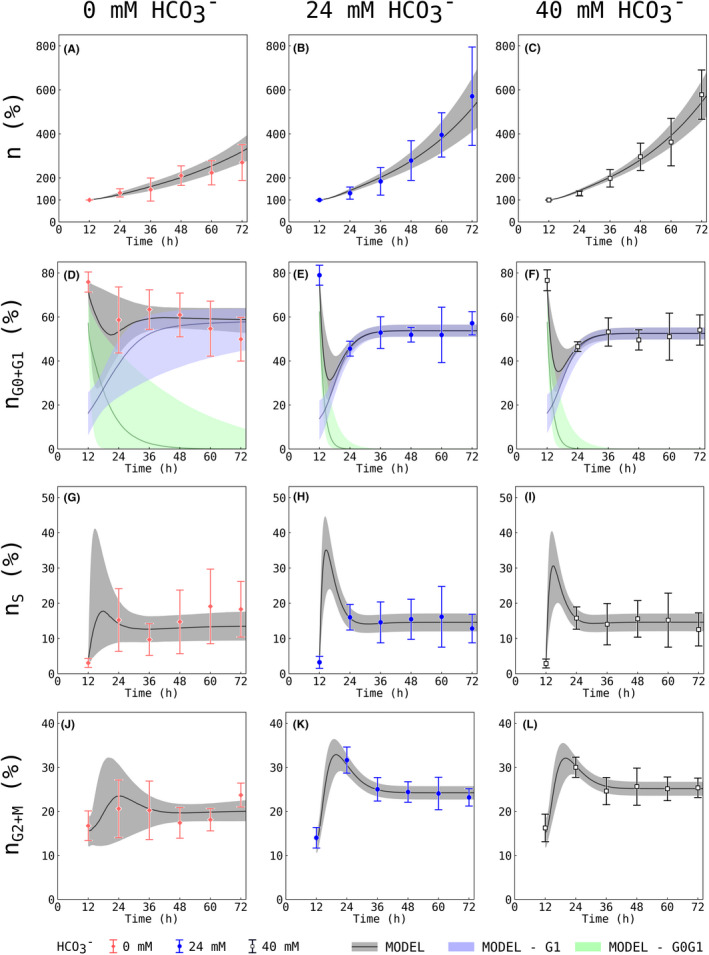
Effect of bicarbonate deprivation on HUVEC experimental data and Bayesian inference. Flow cytometry was used to measure cell number and percentage of cells in the individual cell cycle phases as a function of time for bicarbonate concentrations of 0 (♦, red – A, D, G, J), 24 (●, blue – B, E, H, K) and 40 (□, black – C, F, I, L) mM. Experimental data is shown with mean ± SD. The Bayesian inference‐based model prediction is indicated as black line representing the optimal solution of the differential equations (Equations 1‐5). The light grey areas show the scope between the 10^th^ and the 90^th^ percentile. Light green or light blue plots in panels D‐F indicate cells in either G_0_/G_1_ or G_1_ phase, respectively. The deprivation of bicarbonate leads to changes in the growth patterns and cell cycle dynamics, which are captured by the mathematical model. This data is based on five independent experiments with two replicates in each experiment.

In Figure 5, the estimate of the mathematical model (fitting curve resulting from Bayesian analysis; for parameters see Table 2) is displayed by a dark grey line indicating the median of the mathematical model fit, and a light grey confidence area indicating the 10% and 90% percentiles. Different aspects of the mathematical model are highlighted as follows: First, the mathematical model captures the underlying dynamics of cell proliferation (Figure 5A‐C) and cell cycle for different experimental HCO_3_^−^ concentrations adequately (Figure 5D‐L). Second, the mathematical model allows the distinction of the G_0_/G_1_ and G_1_ phases (cf. green and purple area, Figure 5D‐F) that were not measured separately. Third, the mathematical model predicts peaks in G_0_/G_1_ and S phases (cf. Figure 5 E, F, H, I), which are in between two measurement points at 12 and 24 h. This was not captured by the experimental data, whereas a subsequent smaller and broader peak in G_2_/M phases is also recorded in the data at 24 h (cf. Figure 5K, L).

**TABLE 2:** Bayesian inference and derived parameters for different biological experiments

	Unit	Set A			Set B	
		0 mM HCO_3_^−^	24 mM HCO_3_^−^	40 mM HCO_3_^−^	DMSO	KH7
		Mean ± Bu	Mean ± Bu	Mean ± Bu	Mean ± Bu	Mean ± Bu
Model parameters						
α	(1/h)	0.0461 ± 0.0169	0.0641 ± 0.0093	0.0686 ± 0.0078	0.0606 ± 0.0105	0.1018 ± 0.0948
α_0_	(1/h)	0.2946 ± 0.3240	0.5436 ± 0.2472	0.4873 ± 0.2651	0.4771 ± 0.2859	0.0065 ± 0.004
β	(1/h)	0.1715 ± 0.0523	0.2124 ± 0.0355	0.2222 ± 0.0337	0.2258 ± 0.0417	0.0415 ± 0.0180
γ	(1/h)	0.0898 ± 0.0147	0.0999 ± 0.0119	0.0999 ± 0.0087	0.1146 ± 0.0182	0.0322 ± 0.0144
Mean residence time						
G_1_	(h)	21.7 ± 8.0	15.6 ± 2.3	14.6 ± 1.6	16.5 ± 2.9	9.8 ± 9.1
S	(h)	5.8 ± 1.8	4.7 ± 0.8	4.5 ± 0.7	4.4 ± 0.8	24.1 ± 10.5
G_2_/M	(h)	11.1 ± 1.8	10.0 ± 1.2	10.0 ± 0.9	8.7 ± 1.4	31.1 ± 13.9
G_0_/G_1_	(h)	3.4 ± 3.7	1.8 ± 0.8	2.1 ± 1.1	2.1 ± 1.3	153.8 ± 94.7
G_0_/G_1_ + t_0_	(h)	15.4 ± 3.7	13.8 ± 0.8	14.1 ± 1.1	14.1 ± 1.3	165.8 ± 94.7
Mean cell cycle time						
G_1_ + S + G_2_/M	(h)	38.7 ± 8.3	30.3 ± 2.7	29.1 ± 2.0	29.6 ± 3.3	65.0 ± 19.6
G_0_/G_1_ + t_0_ + S + G_2_/M	(h)	32.4 ± 4.5	28.6 ± 1.7	28.6 ± 1.6	27.3 ± 2.0	221.0 ± 96.3
Mean doubling time						
Period: 24 h‐72 h	(h)	36.2 ± 4.5	26.1 ± 3.1	25.0 ± 2.2	25.6 ± 3.8	90.0 ± 67.8

*Note*: Model parameters are shown as mean with Bayesian uncertainty (bu) of the estimated posterior functions. In the case of a normal distributed posterior, Bayesian uncertainty would be equal to the standard deviation, which in this case reflects the biological scattering (cf. Figure 4). Mean residence times are calculated as reciprocals of model parameters as described above using error propagation to obtain correct uncertainties. The variable t_0_ = 12 h accounts for the period between starvation and the first measurement point.

#### 3.3.2 Model parameters enable quantitative comparisons between different HCO_3_ ^−^ concentrations

The characteristics of the model fits could be quantified by parameter values (cf. Appendix S1: Supplement S5, Table ST1). Parameters were converted into mean residence times, which represent the mean times cells spent in individual phases (Section 2.2). A difference was found for mean cell cycle time (G_1_ + S + G_2_/M) between cells exposed to 0 and 24 mM HCO_3_^−^ (38.7 ± 8.3 h vs. 30.3 ± 2.7 h). However, no difference was found between 24 and 40 mM HCO_3_^−^. The difference of mean cell cycle times of about 8.4 h between 0 and 24 mM HCO_3_^−^ can be attributed to a delay of 6.1 h of G_1_ phase (21.7 ± 8.0 h vs. 15.6 ± 2.3 h) and 1.1 h of S phase (5.8 ± 1.8 h vs. 4.7 ± 0.8 h) as well as 1.1 h of G_2_/M phases (11.1 ± 1.8 h vs. 10.0 ± 1.2 h). According to the parameter estimation, the withdrawal of the endogenous ADCY10 activator bicarbonate resulted in a prolongation of cell cycle times with a major delay of G_1_ phase.

### 3.4 Model parameters reveal the effect of the selective pharmacological ADCY10 inhibitor KH7 on the progression of individual cell cycle phases

**FIGURE 6:**
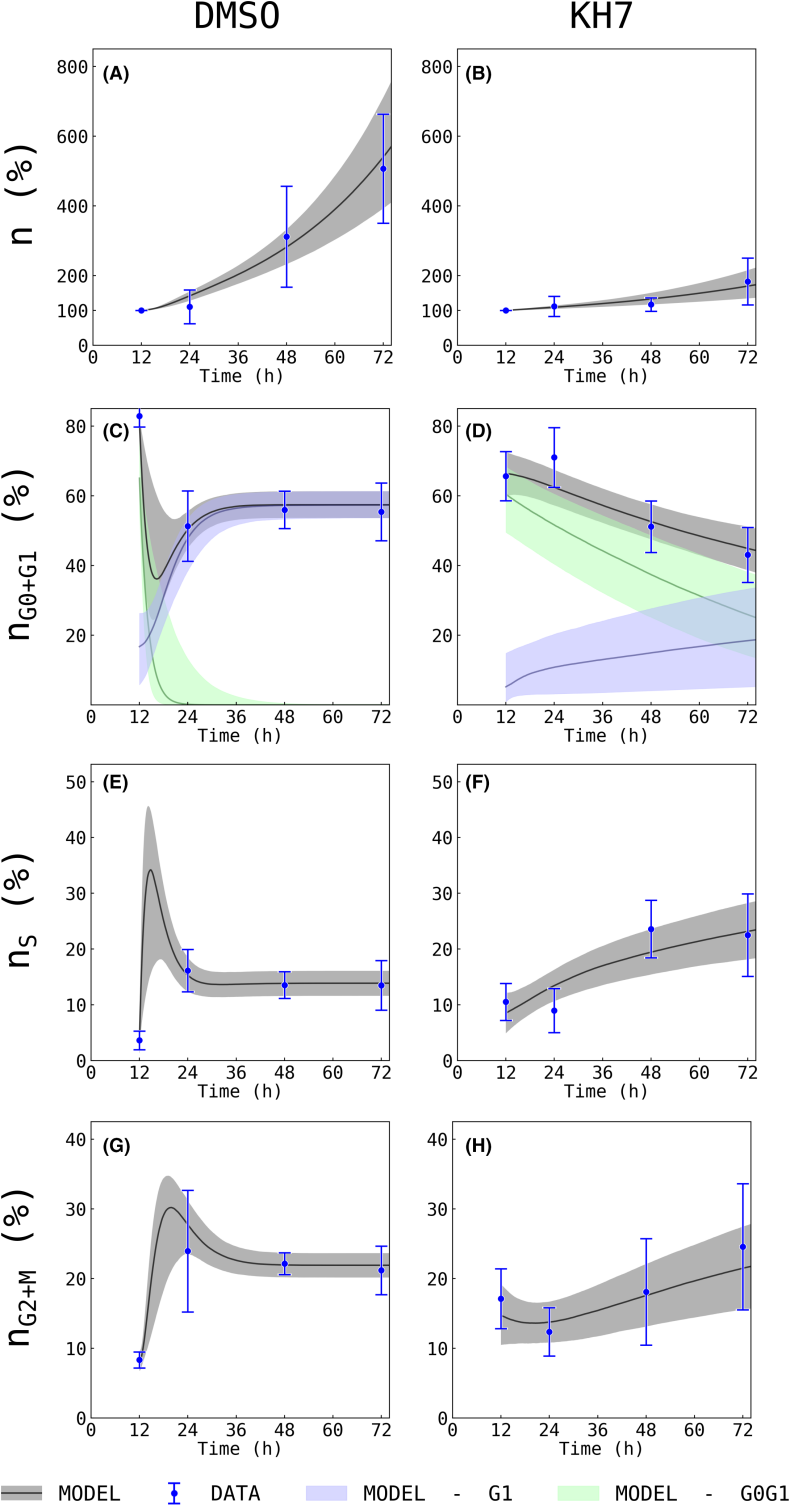
Effect of KH7 on experimental data and Bayesian inference. Flow cytometry was used to measure cell number and percentage of cells in the individual cell cycle phases as a function of time in the presence of KH7. DMSO served as control. Experimental data is shown in blue with mean ± SD. The Bayesian model prediction is indicated as black line. The light grey areas show the scope between the 10^th^ and the 90^th^ percentile. Light green or light blue plots in panels C and D indicate cells in either G_0_/G_1_ or G_1_ phase, respectively. The addition of KH7 leads to changes in the growth patterns and cell cycle dynamics, which are captured by the mathematical model. This data is based on five independent experiments with two replicates in each experiment.

During both, set A (analysing the effect of HCO_3_^−^ on cell cycle) and set B (analysing the effect of KH7 on the cell cycle), a control group representing a physiological state was established. This was 24 mM HCO_3_^−^ in set A and DMSO in set B. The estimated parameters for control groups of both experimental sets were in good agreement (cf. Appendix S1: Supplement S5, Table ST1, 24 mM vs. DMSO). Thus, DMSO in the used concentration of 10 μM seems to have no influence on cell cycle phases. However, model parameters indicated dramatic changes in cell cycle progression under the influence of KH7 (see Figure 6). The mean, which was corrected for the variable t_0_ (=12 h), residence time of G_0_/G_1_ phase was prolonged from 14.1 ± 1.3 h to 165.8 ± 94.7 h (cf. Table 2). Keeping in mind that the Bayesian uncertainty represents the biological variability rather than measurement errors (cf. Appendix S1: Supplement S12, Figure SF2), this result does indicate both a prolongation and an increase in heterogeneity of cellular behaviour. Further, parameters indicate a prolongation of mean residence time in S phase by about 5.4‐folds and G_2_/M phase by about 3.6‐folds. In this context, the mean duration of G_1_ phase has to be interpreted with care, since the doubling time and the mean duration of cells remaining in G_0_/G_1_ exceeded the length of the experiment. Hence, the majority of the cells never finished a full cell cycle during the time of the experiments and never reached G_1_ phase.

Taken together, the ADCY10 inhibitor KH7 prevented HUVECs from leaving G_0_ phase after cell starvation. Furthermore, KH7 caused an increase in mean cell cycle time and prolonged G_0_/G_1_, S and G_2_/M to such a degree that it resembles a functional cell cycle arrest.

## 4 DISCUSSION

Endothelial angiogenesis is known to be driven by external stimuli such as VEGF, TGF, TNF‐α, PGF and others. These factors trigger cascades of second messengers like cAMP, cGMP, Ca^2+^, IP_3_ and DAG via interactions with membrane‐associated receptors. By contrast, the soluble ADCY10 is expressed in a variety of cell compartments, thus displaying an alternative cAMP source compared with the pathways mentioned above. This study delivers the first evidence that bicarbonate‐activated ADCY10 plays a significant role in cell cycle control in human endothelial cells. Endothelial cells express the ADCY‐isoforms 4, 5 and 6.^47^, ^48^ Ca^2+^ acts as an inhibitor on ADCY5 and 6, while it has no effect on ADCY4. By contrast, ADCY10 can be activated by Ca^2+^ independent of calmodulin.^49^ Reportedly, ADCY10 activity is linked to the Ca^2+^ homoeostasis of the cell, in which Ca^2+^ and HCO_3_^−^ can act synergistically on ADCY10 activity.^49^, ^50^ Therefore, sole existence of calcium in culture media may as well influence ADCY10 activity and leads to increased cell proliferation. Despite the culture media used throughout this study contained full supplemented growth factors (EGF, ECGS, HC and FGF) and serum, inhibition of ADCY10 led to suppressed cell proliferation. This result suggests that ADCY10 can act as an endogenous angiogenic mediator, which indicates a fundamental role of ADCY10 in cell and tissue culture.

Even though physiological bicarbonate blood levels are in the range of 22–26 mM in serum of mammals, bicarbonate levels of intracellular subcompartments are assumed to be highly dynamic.

While CO_2_ is a conjugate acid that can diffuse freely across membranes, HCO_3_^−^ as it is charged relies on transport proteins such as CFTR, CaCC, AE2 and NBCe1^51^ to maintain the homoeostasis of an intracellular bicarbonate concentration at around 10 mM under physiological condition. It is reported that bicarbonate levels can drop to levels of 3 mM under metabolic load.^52^ This physiological range is close to the EC50 of isolated ADCY10 at around 12 mM HCO_3_^−^.^53^ This is in good accordance with the current study showing no differences between 24 and 40 mM HCO_3_^−^ due to HCO_3_^−^ saturation. Despite the global intracellular bicarbonate concentration, the exact levels of bicarbonate of the intracellular subcompartments are beyond the scope of our available measurement technique. Keeping the complex mechanisms of bicarbonate transport in mind, we designed clearly defined experimental conditions of extracellular bicarbonate using total deprivation (0 mM), physiological (24 mM) or supraphysiological (40 mM) concentrations. However, concentrations of intracellular subcompartments are known to differ^54^, ^55^ and might play a role in the fine‐tuning and the orchestration of cell cycle progression.

The experimental usage of the ubiquitous HCO_3_^−^ inevitably raises the question, to which extent other pathways might be affected by changes in the HCO_3_^−^ milieu. While we cannot rule out any unknown factor, looking at known biochemical enzymatic reactions might shed some light into the dark: During the synthesis of the DNA/RNA building block UMP, carbamoyl‐phosphate synthetase II is dependent on bicarbonate with a Km value of only 1.4 mM, which is sevenfold below the physiological intracellular HCO_3_^−^ concentration.^56^ Similar small Km values <2 mM for bicarbonate are described for vitamin K carboxylations.^57^, ^58^ Hence, ADCY10 is regulated by physiological HCO_3_^−^ concentrations, while general carboxylases are mainly unaffected due to their low Michaelis–Menten constant.

ADCY10 may influence cell cycle control via cellular metabolism. ADCY10 is known to be the only local source of cAMP in mitochondria matrix.^59^ In fact, ADCY10 has been identified to control mitochondrial ATP production.^7^, ^10^, ^11^ ATP production during G_1_ phase is key for a physiological G_1_‐S transition. Nutrient deprivation by serum withdrawal induces cell cycle arrest in G_0_/G_1_.^60^ However, it remains unclear, whether ADCY10 inhibition leads to prolongation of G_1_ phase by pure metabolic function or via cAMP‐dependent regulation of cell cycle checkpoints.^61^

Our assumptions about cell cycle progression are formalized by the mathematical model. The analysis showed that Bayesian inference could quantify experimental data on cell cycle progression in terms of model parameters for residence times of individual cell cycle phases. Furthermore, it predicted the continuous temporal dynamics interpolating between measured biological experimental data points. In contrast to the traditionally used mean doubling time based on the assumption of pure exponential growth, the model parameters allow a more specific differentiation of the underlying cell cycle progression. A doubling time‐based approach is generally limited to cell populations at steady state. By contrast, the here proposed formalism allows to analyse cell populations at steady state as well as synchronized cell populations. While doubling time describes how long an entire cell population needs to double its number, this is not the same as the average time an individual cell needs to complete one full cell cycle (see Section 3.2). Generally, the choice of whether doubling time or mean cell cycle time should be used depends on the underlying research question. In this particular case focussing on cell cycle duration, the mean cell cycle time should be favoured.

Established methods to assess cell cycle dynamics include measurements of DNA content dye, measurements of thymidine analogues, time course of cell counting, time‐lapse microscopy and fluorescent dye retention assays.^61^ Furthermore, Western blot analysis can identify cell cycle‐specific markers such as cyclins. However, some of these methods might cause cellular toxicity and therefore lead to cell cycle alteration. In addition, these approaches are limited in terms of identifying individual cell cycle stages. The method introduced here allows us to combine the information of time course measurements of DNA content as well as time course measurements of cell numbers into one combined approach using mathematical modelling. While FACS analysis is commonly available, there is no extra computer hardware needed as calculations can be performed on virtually any standard desktop computer. Bayesian inference allows to consistently integrate the complete information of time course studies—which is commonly either only graphically presented or projected into a parameter that combines start and endpoint. Bayesian inference was used to link experimental data to a cell cycle model. Even though the model is a simplified abstraction of the overall complex cell cycle dynamics of the real world—it represents a quantitative description of this process. One step further, such a model can be solved in theory (or numerically) and can therefore be used for predictions: Either in experimental planning or in special cases where experimental quantities are hard to assess or even out of scope of available measurement techniques. The linkage between the real world and mathematical model description is intrinsic to Bayesian inference and provides a powerful complement to statistical comparisons of data of different groups. Access to our simulation code is provided via zenodo.^37^

In conclusion, we were able to identify and specify the role of bicarbonate‐activated ADCY10 as a key regulator in cell cycle progression with influence on all phases of the cell cycle and G_1_ in particular. We showed that our combined Bayesian approach including mathematical modelling, in silico experimental design and in vitro data allowed to facilitate quantitative data interpretation and to optimize workload and required resources for experiments. With regard to the biological implications of this work, the analysis of the role of the metabolic sensor ADCY10 as a potential therapeutic option remains to be elucidated. One may speculate that targeted orchestration of ADCY10 under conditions of impaired cell metabolism and endothelial regenerative dysfunction, as found, for example, in diabetes mellitus may become a possible therapeutic option.

## AUTHOR CONTRIBUTIONS

**Warunya Woranush:** Conceptualization (equal); data curation (equal); formal analysis (equal); investigation (equal); methodology (equal); visualization (equal); writing – original draft (equal); writing – review and editing (equal). **Mats Leif Moskopp:** Conceptualization (equal); data curation (equal); formal analysis (equal); investigation (equal); methodology (equal); software (equal); visualization (equal); writing – original draft (equal); writing – review and editing (equal). **Thomas Noll:** Conceptualization (equal); investigation (equal); supervision (equal); validation (equal); writing – original draft (equal); writing – review and editing (equal). **Peter Dieterich:** Conceptualization (equal); investigation (equal); supervision (equal); validation (equal); writing – original draft (equal); writing – review and editing (equal).

## CONFLICT OF INTEREST

The authors declare that no conflicts of interest exists.

## Supporting information


Appendix S1

## Data Availability

The data that support the findings of this study are openly available in the following repository: Woranush W, Moskopp ML, Noll T, Dieterich P. Supplementary data of “ADCY10 is a key regulator of cell cycle control.” doi:10.5281/zenodo.5147985.
