# Supplementary material for: Quantifying and mathematical modelling of the influence of soluble adenylate cyclase on cell cycle in human endothelial cells with Bayesian inference
Source: J Cell Mol Med. 2022 Nov 13;26(23):5887–900. doi: 10.1111/jcmm.17611 (PMC9716222; doi:10.1111/jcmm.17611)
Supplement: Supplementary file 1 — Appendix S1 [file JCMM-26-5887-s001.docx]

**SUPPLEMENT**

**S1. Ethics approval**

Ethics approval was granted by the ethics committee of the Medizinische Fakultät Carl Gustav Carus of the Technische Universität Dresden (permission EK 203112005).

**S2. Ordinary differential equations of the cell cycle model for relative numbers**

In the main text of this article Eqs. 1 – 5 represent a system of differential equations for absolute numbers. However, here we give a converted system of non-linear differential equations describing the percentages of cells per phase:

$\frac{d}{dt}n_{G0,G1}\left( t \right)=-\alpha_{0}\times n_{G0,G1}\left( t \right)-\gamma\times n_{G2,M}\left( t \right)\times n_{G0,G1}\left( t \right)$ (Eq. SE1)

$\frac{d}{dt}n_{G1}\left( t \right)=2\times\gamma\times n_{G2,M}\left( t \right)-\alpha\times n_{G1}\left( t \right)- \gamma\times n_{G2,M}\left( t \right)\times n_{G1}\left( t \right)$ (Eq. SE2)

$\frac{d}{dt}n_{S}\left( t \right)=\alpha\times n_{G1}\left( t \right)+\alpha_{0}\times n_{G0,G1}\left( t \right)-\beta\times n_{S}\left( t \right)- \gamma\times n_{G2,M}\left( t \right)\times n_{S}\left( t \right)$ (Eq. SE3)

$\frac{d}{dt}n_{G2,M}\left( t \right)=\beta\times n_{S}\left( t \right)-\gamma\times n_{G2,M}\left( t \right)- \gamma\times n_{G2,M}\left( t \right)\times n_{G2,M}\left( t \right)$ (Eq. SE4)

$\frac{d}{dt}N\left( t \right)=\gamma\times n_{G2,M}\left( t \right)\times N\left( t \right)$ (Eq. SE5)

These equations are helpful in studying the steady state, where the fractions of cells in each state are constant. In addition, Eq. SE5 shows pure exponential growth under steady state conditions with a doubling time of $\ln\left( 2 \right)/(\gamma\times\bar{n_{G2,M}})$where $\bar{n_{G2,M}}$ is the corresponding steady state fraction of cells.

**S3. Unclassified count estimator U**

The unclassified count estimator U is used to record the unclassified counts of flow cytometry (cf. Figure 1B and Section 2.2). The core implementation of the mathematical model uses absolute numbers. To fit simulations to biological data, absolute numbers of the simulation (e.g. N_G1_) of the cell cycle stages are converted as illustrated exemplarily for n_G1_:

$n_{G1}\left( t \right)=\frac{N_{G1}\left( t \right)}{N\left( t \right)}*\left( 1-U \right)$ (Eq. SE6)

**S4. Agent-based model adaptation**

To demonstrate the generalizability of the here introduced approach, an agent-based model of the cell cycle was implemented. Cells are represented by agents. Agents are in one of four states (G_0_/G_1_, G_1_, S or G_2_/M). Individual waiting times *t_w,i_ ≥ 0* per phase are drawn from exponential distributions with probability density functions in the form of $pdf\left( t_{w,i},1/\bar{\alpha} \right)=\bar{\alpha}\times\exp\left( -t_{w,i}\times\bar{\alpha} \right)$, where the scale parameter is based on the reciprocal values of the means of transition rates ($\bar{\alpha}$, $\bar{\alpha_{0}}$, $\bar{\beta}$, $\bar{\gamma}$) of the differential equation Eq. 1 – 5. The results are in good accordance with those of the differential equations (Figure SF1). Thus, our continuous mass-action law similar model leads to the same result as the individual stochastic cell simulation, which underlines the universality of this approach.


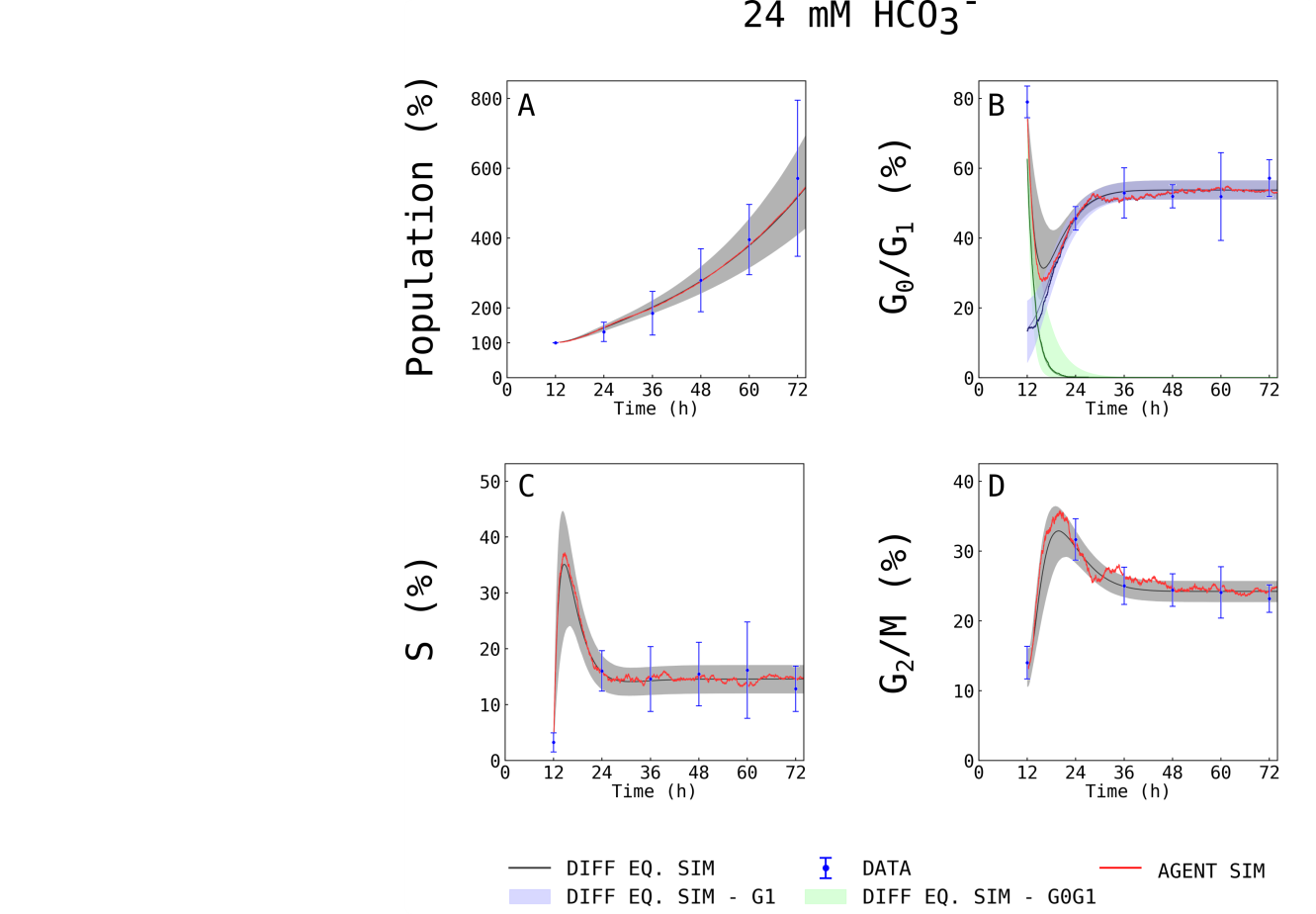


**FIGURE SF1 Agent-based model adaptation of the differential equation system.** N = 1000 agents were used. The time interval of the simulation steps was dt = 30 s (≈ 0.0083 h). Agent-based result red, solution of Eq.1-5 in grey with median, 10^th^ and 90^th^ percentile, biological experimental data is shown in blue with mean and standard deviation.

**S5. Parameter estimation**

Table ST1 shows the results of the parameter estimation from Bayesian data analysis.

**TABLE ST1 Estimated parameters for the individual sets of experiments.**

**S6. Numerical implementation of Bayesian inference**

Here, we use the Python interface pymultinest of the multinested extension of the nested sampling algorithm^44^, as a numerical implementation of Bayesian inference.^39–43^

This algorithm performs a step wise search for parameter sets with the highest likelihoods in the given prior-likelihood space of the free model parameters (Table 1). Initially parameter sets with random parameter values are scattered within the prior space. Each of these initial parameter sets is called a *live point* in nested sampling terminology. Here, we used 1000 live points. All live points are evaluated for their likelihood. The likelihood is a measure of consistency between experimental data and the values of the mathematical model based on a specific parameter set. The difference between experimental data and model values is weighted by the measurement uncertainty of the data (see Eq. 8). In a step wise process, the live point with the lowest likelihood is replaced by a new one with a higher likelihood. This leads to condensation of the live points in regions of parameter sets with high likelihood. Finally, the posterior probability for the individual parameter sets can be calculated by numerical integration and marginalization of the traces of replaced live points throughout the sampling process. A more detailed mathematical description of this algorithm is beyond the scope of this article, but can be found in the original articles.^39–43^

**S7. Hardware and calculation times**

After code optimization parameter estimation for single sets of real experimental data was performed on a standard desktop computer (Intel^®^ Core^TM^ i5-3320M CPU, 8 GB Ram) with typical calculation times in the range of 3 – 5 minutes and memory usage below 100 MB.

**S8. Results of statistical significance testing**

**TABLE ST2 Synthetic test data values to mimic HUVEC cell cycle after starvation.**

| **1way ANOVA Testing** | | | |
| --- | --- | --- | --- |
| Parameter | $\boldsymbol{\alpha}$ |  |  |
| One-way analysis of variance |  |  |  |
| P value | 0.0276 |  |  |
| P value summary | * |  |  |
| Newman-Keuls Multiple Comparison Test | Mean Diff. | P < 0.05? | Summary |
| 0 mM vs 24 mM | -0.018 | Yes | * |
| 0 mM vs 40 mM | -0.0225 | Yes | * |
| 24 mM vs 40 mM | -0.045 | No | ns |
|  |  |  |  |
| Parameter | $\boldsymbol{\alpha}_{\boldsymbol{0}}$ |  |  |
| One-way analysis of variance |  |  |  |
| P value | 0.3698 |  |  |
| P value summary | ns |  |  |
| Newman-Keuls Multiple Comparison Test | Mean Diff. | P < 0.05? | Summary |
| 0 mM vs 24 mM | -0.2490 | No | ns |
| 0 mM vs 40 mM | -0.1927 | No | ns |
| 24 mM vs 40 mM | -0.0563 | No | ns |
|  |  |  |  |
| Parameter | $\boldsymbol{\beta}$ |  |  |
| One-way analysis of variance |  |  |  |
| P value | 0.1635 |  |  |
| P value summary | ns |  |  |
| Newman-Keuls Multiple Comparison Test | Mean Diff. | P < 0.05? | Summary |
| 0 mM vs 24 mM | -0.0409 | No | ns |
| 0 mM vs 40 mM | -0.0507 | No | ns |
| 24 mM vs 40 mM | -0.0098 | No | ns |
|  |  |  |  |
| Parameter | $\boldsymbol{\gamma}$ |  |  |
| One-way analysis of variance |  |  |  |
| P value | 0.3414 |  |  |
| P value summary | ns |  |  |
| Newman-Keuls Multiple Comparison Test | Mean Diff. | P < 0.05? | Summary |
| 0 mM vs 24 mM | -0.0101 | No | ns |
| 0 mM vs 40 mM | -0.0101 | No | ns |
| 24 mM vs 40 mM | 0.0000 | No | ns |

**S9. Synthetic test data generation**

Synthetic test data was simulated by using the mathematical model and a fixed set of parameters (Table ST3). Parameter values were based on the experimenters’ general experiences to match HUVEC cell cycle after synchronization. This allows to test whether Bayesian inference is capable of correct parameter estimation for a synthetic test data set with known parameter values (Table ST3). Seven different sets of test data were simulated for sampling intervals dt of 1, 2, 4, 6, 12, 24, or 36 h and a total observation period of 72 h.

**TABLE ST3 Parameters of synthetic test data values to mimic HUVEC cell cycle after starvation.**

| **Name** | | **Unit** | **Test Value** |
| --- | --- | --- | --- |
| **Start conditions** | |  |  |
| n_G1_(t_0_) | | (%) | 10 |
| n_G0,G1_(t_0_) | | (%) | 75 |
| n_S_(t_0_) | | (%) | 5 |
| n_G2,M_(t_0_) | | (%) | 10 |
|  | |  |  |
| **Model parameters** | |  |  |
| α | (G_1_ → S) | (1/h) | 1 / 16 |
| α_0_ | (G_0_G_1_ → S) | (1/h) | 1 / 2 |
| β | (S → G_2_,M) | (1/h) | 1 / 5 |
| γ | (G_2_,M → G_1_) | (1/h) | 1 / 10 |
|  | |  |  |
| **Unclarified count estimator** | |  |  |
| U | | (%) | 5 |

**S10. Measurement error of the synthetic test data**

In the last step of test data generation, Gaussian noise was added to the data to mimic the influence of measurement errors. A relative Gaussian noise of σ_cell number_ = 20% was added to the synthetic data points representing cell number as well as a noise of σ_flow cytometry_ = 5%, which was added as percentage to data points representing cell cycle phases.

**S11. Calculation of the doubling time.**

The calculation of the doubling time and its standard deviation is based on cell numbers from the mathematical model in the time period between 24 and 72 h and would only be true for exponential growth (cf. Table 2). The mean doubling time t_D_ was generally calculated according to the following equation:

$t_{D}=\frac{{(t}_{2}-t_{1})}{{log}_{2}\left( \frac{N\left( t_{2} \right)}{N\left( t_{1} \right)} \right)}$ (Eq. SE7)

With t_2_ = 72 h and t_1_ = 24 h. It has to be pointed out that the assumption of exponential growth in cell culture is often chosen by life science experimenters despite its considerable error especially for small quantities of synchronized cells or limited growth capacity of the experimental system.

**S12. Combined analysis of independent experiments**

After the proof of principal on simulated test data (section 3.2 of the main text), data analysis was performed with real experimental data. For each condition (0, 24 or 40 mM HCO_3_^-^) five independent experiments were conducted, each one consisting of two replicates of the same source of HUVEC cells. Two analyses were performed: In the first set, each independent experiment was analysed using the here proposed method. In the second set, the data points of all five experiments were pooled together and the proposed method was applied to the pooled data in order to estimate cell cycle parameters. Results of the data analysis for each of the five individual experiments and the pooled group of all experiments are exemplarily shown in Figure SF2. As can be seen by the scattering of the individual data points, the biological data shows a relevant degree of biological scattering. This puts the error bars of the group analysis into perspective as they reproduce the biological heterogeneity. Therefore, the grouped data was used for further analysis throughout this work.


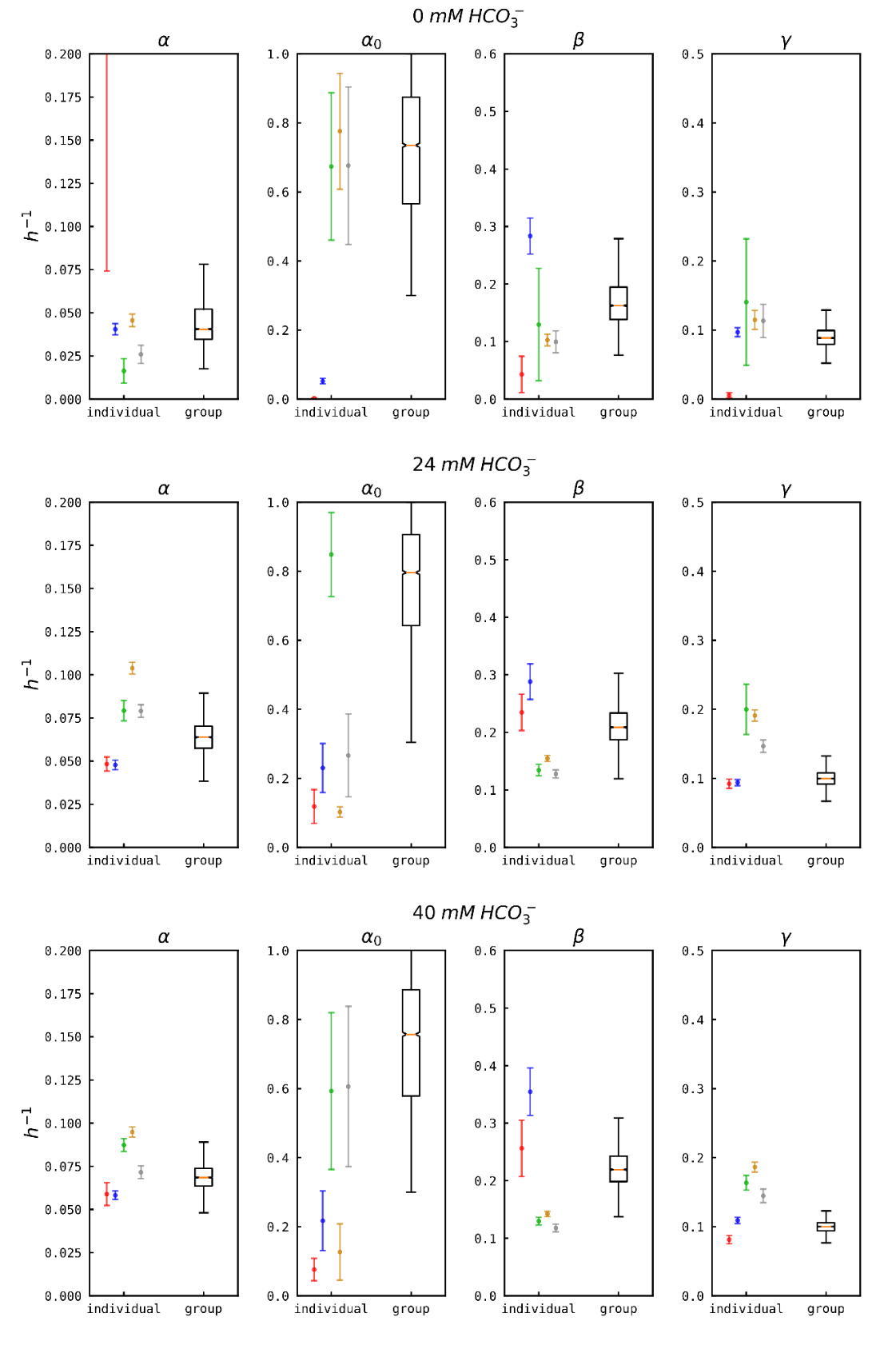


**FIGURE SF2 Analysis of individual and grouped data.** Parameter estimation was performed for either independent experiments or the pooled data (group) for 0, 24 and 40 mM HCO_3_^-^. Independent experiments showed a biological heterogeneity. Overall biological heterogeneity was covered by the analysis of grouped data. Individual data sets are plotted as mean plus/minus standard deviation. Box-whisker plots indicate median (orange), 25^th^ and 75^th^ percentile (box) as well as 1.5 times interquartile distance of 25^th^ to 75^th^ percentile (whisker).
